# Acetate Metabolism in Anaerobes from the Domain *Archaea*

**DOI:** 10.3390/life5021454

**Published:** 2015-06-09

**Authors:** James G. Ferry

**Affiliations:** Department of Biochemistry and Molecular Biology, Pennsylvania State University, University Park, PA 16802, USA; E-Mail: jgf3@psu.edu; Tel.: +1-814-863-5721

**Keywords:** methanogenesis, fermentation, respiration, *Methanosarcina*, *Pyrococcus*, carbon monoxide

## Abstract

Acetate and acetyl-CoA play fundamental roles in all of biology, including anaerobic prokaryotes from the domains *Bacteria* and *Archaea*, which compose an estimated quarter of all living protoplasm in Earth’s biosphere. Anaerobes from the domain *Archaea* contribute to the global carbon cycle by metabolizing acetate as a growth substrate or product. They are components of anaerobic microbial food chains converting complex organic matter to methane, and many fix CO_2_ into cell material via synthesis of acetyl-CoA. They are found in a diversity of ecological habitats ranging from the digestive tracts of insects to deep-sea hydrothermal vents, and synthesize a plethora of novel enzymes with biotechnological potential. Ecological investigations suggest that still more acetate-metabolizing species with novel properties await discovery.

## 1. Introduction

Acetate and acetyl-CoA play a prominent role in the metabolism of all three phylogenetic domains of life, including anaerobic prokaryotes from the domains *Bacteria* and *Archaea*, which contribute to an estimated quarter of all living protoplasm in Earth’s biosphere [1]. Anaerobes from the domain *Archaea* play significant roles in the global carbon cycle by metabolizing acetate as a growth substrate or product. Many anaerobes also fix CO_2_ into cell material via synthesis of acetyl-CoA [2]. 

**Figure 1:**
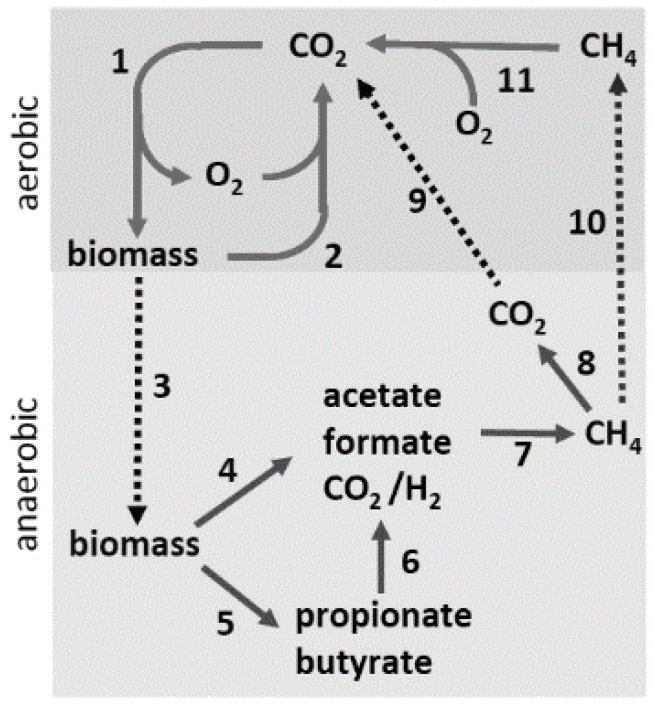
The global carbon cycle. Solid lines indicate steps in the cycle (see text) and dotted lines indicate transfer of material between aerobic and anaerobic environments.

In anaerobic environments where terminal electron acceptors (Fe(III), Mn(IV), SO_4_^2−^, S^0^, NO_3_^−^) are absent, anaerobes from both prokaryotic domains convert complex organic matter to CH_4_ and CO_2_, providing an essential link in the global carbon cycle (Figure 1). In Step 1, CO_2_ is incorporated into biomass driven primarily by photosynthesis. In environments where O_2_ is abundant, microbes oxidize the biomass, producing CO_2_ that re-enters the carbon cycle (Step 2). A significant portion of the biomass is diverted to an assortment of anaerobic habitats devoid of terminal electron acceptors (Step 3), where anaerobic microbial food chains, comprised of at least three distinct metabolic groups, digest the biomass to CO_2_ and CH_4_ (Steps 4–7). The fermentative group converts the complex biomass primarily into, acetate along with lesser amounts of volatile fatty acids, H_2_, and CO_2_ (Steps 4 and 5). The fatty acids are converted chiefly into acetate plus either formate or H_2_ by the acetogenic group (Step 6). Thus, acetate emerges as the principal product of the fermentative and acetogenic groups. The CH_4_-producing (methanogen) group is sub-divided into acetate-utilizing (acetoclastic) and CO_2_-reducing species. Acetoclastic species convert the methyl group of acetate to CH_4_ and the carbonyl group to CO_2_. The CO_2_-reducing species reduce CO_2_ to CH_4_ with electrons derived from H_2_ or formate. At least two-thirds of the CH_4_ produced derives from acetate, the central intermediary in anaerobic microbial food chains. A portion of the CH_4_ is oxidized to CO_2_ (Step 8) by associations of anaerobes that utilize sulfate, nitrate, manganese, or iron as terminal electron acceptors [3]. The CO_2_ and remaining CH_4_ diffuse into aerobic zones (Steps 9 and 10), where O_2_-requiring methanotrophic microbes oxidize the CH_4_ to CO_2_ (Step 11), completing the carbon cycle. Anaerobes also participate in chemoautotrophic habitats, where they fix carbon dioxide in catabolic and anabolic pathways. Although the fermentative and acetogenic groups are largely populated with characterized isolates from the domain *Bacteria*, all characterized methanogens are classified in the domain *Archaea*. Acetate-utilizing anaerobes classified in the domain *Archaea* also proliferate in environments where terminal electron acceptors are abundant and obtain energy through anaerobic respiration, converting acetate to CO_2_.

This review encompasses the role of acetate, which has the greatest influence on the ecology of environments, in the energy conversion pathways of anaerobes from the domain *Archaea*; however, acetate and acetyl-CoA also play a prominent role in the biosynthetic pathways of anaerobes from the domain *Archaea* [2].

## 2. Acetate Production

### 2.1. Heterotrophic Energy-Converting Pathways Producing Acetate

**Figure 2:**
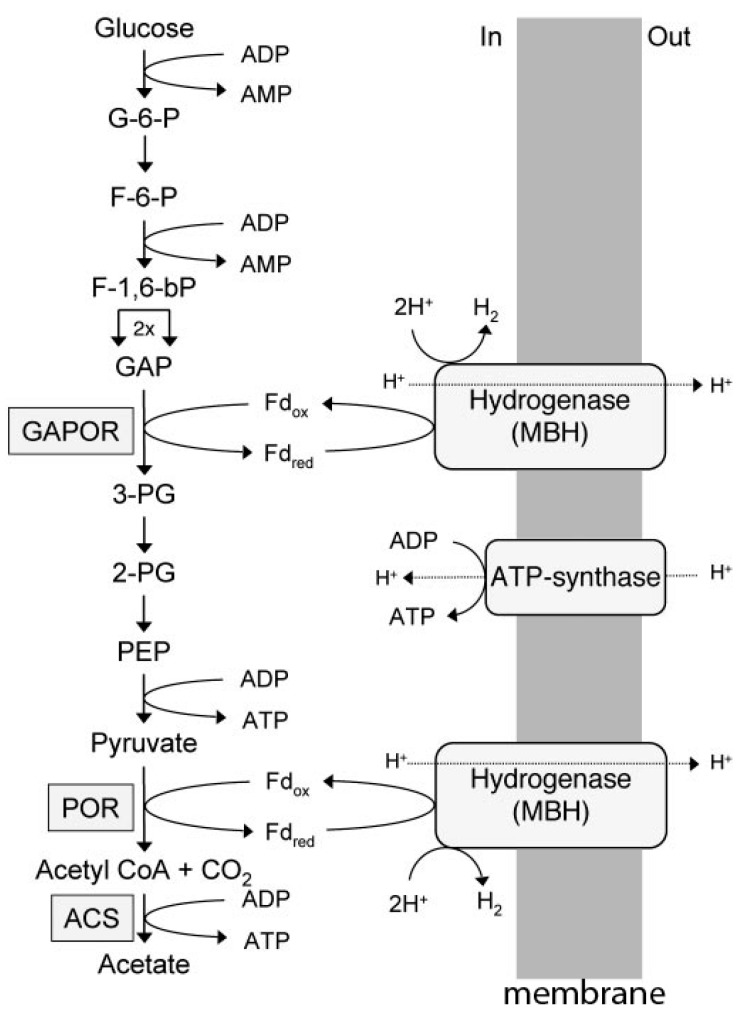
Electron transport and energy conversion during glucose catabolism by *P. furiosus*. G-6-P, glucose 6-phosphate; F-6-P, fructose 6-phosphate; F-1,6-bP, fructose 1,6-bisphosphate; GAP, glyceraldehyde phosphate, GAPOR, GAP: Ferredoxin oxidoreductase; 3-PG, 3-phosphoglycerate; 2-PG, 2-phosphoglycerate; PEP, phosphoenolpyruvate; POR, pyruvate: Ferredoxin oxidoreductase; ACS, acetyl-CoA synthase. Reproduced with permission [4]. Copyright (2006) National Academy of Sciences, USA.

Investigations of heterotrophic hyperthermophilic species from the domain *Archaea* have revealed pathways that deviate substantially from pathways in heterotrophic organisms from the domain *Bacteria*. *Pyrococcus furiosus*, for example, grows at 100 °C and ferments carbohydrates to acetate, CO_2_, and H_2_ by an unusual Emden–Meyerhof pathway involving the novel enzymes ADP-dependent glucokinase, ADP-dependent phosphofruktokinase, glyceraldehyde-3-phosphate ferredoxin oxidoreductase (GAPOR), phosphoenolpyruvate synthase, pyruvate: Ferredoxin oxidoreductase (POR), and ADP-forming acetyl-CoA synthetase (Figure 2) [4]. Other heterotrophic acetate-producing hyperthermophiles that utilize one or more of these enzymes include species from the genera *Thermococcus*, *Desulfurococcus*, *Staphylothermus*, and *Archaeoglobus* [5,6,7,8]. In the glycolytic pathway exemplified by *P. furiosus* (Figure 2), ferredoxin reduced by GAPOR and POR is re-oxidized by a membrane-bound hydrogenase that generates an ion gradient driving ATP synthesis [4]. GAPOR is an oxygen-sensitive homomonomer with a molecular mass of 63 kDa, and contains a pterin cofactor, one tungsten, and six iron atoms per monomer [9]. Pyruvate is oxidized to acetyl-CoA, catalyzed by pyruvate: Ferredoxin oxidoreductase (POR). The enzyme has a molecular mass of 100 kDa and is comprised of three subunits (45, 31, and 24 kDa), and contains thiamine pyrophosphate (TPP) and two ferredoxin-type [4Fe-4S] clusters [10]. The enzyme requires CoASH but not TPP for pyruvate oxidation activity. The POR also catalyzes the formation of acetaldehyde from pyruvate in a CoA-dependent reaction, although the cofactor plays a structural rather than catalytic role [11]. Acetyl-CoA is converted to acetate by an ADP-forming acetyl-CoA synthetase, which generates ATP by substrate level phosphorylation [12,13]. Conversion of acetyl-CoA to acetate by acetyl-CoA synthetases is characteristic of the domain *Archaea*, in contrast to the domain *Bacteria*, in which phosphotransacetylase and acetate kinase predominate [14]. A reaction mechanism has been proposed for the heterotetrameric (α_2_β_2_) enzyme from *P. furiosus* that follows a four-step mechanism including transient phosphorylation of two active site histidine residues (Equations (1)–(4)) [15].

(1) *E* + acetyl-CoA + Pi = *E*·acetyl~P + CoASH

(2) *E*·acetyl~P = acetate + *E*-His^257^α~P

(3) *E*-His^257^α~P = *E*-His^71^β~P

(4) *E*-His^71^β~P + ADP = ATP + *E*

*Archaeoglobus fulgidus* is an example of a heterotrophic hyperthermophile from the domain *Archaea* that utilizes terminal electron acceptors in place of reducing protons and producing H_2_. A strain of *A. fulgidus* grows with starch as the sole source of carbon and energy by an incomplete oxidation of glucose to acetate and CO_2_, utilizing a modified Embden–Meyerhof pathway resembling that of *P. furiosus* [8]. However, in contrast to H_2_-producing *P. furiosus*, electrons derived from the oxidation are transferred to sulfate, producing sulfide (Equation (5)):

(5) C_6_H_12_O_6_ + H_2_SO_4_ = 2CO_2_ + 2C_2_H_4_O_2_ + H_2_S + 2H_2_O.

### 2.2. Chemolithotrophic Energy-Converting Pathways Producing Acetate

**Figure 3:**
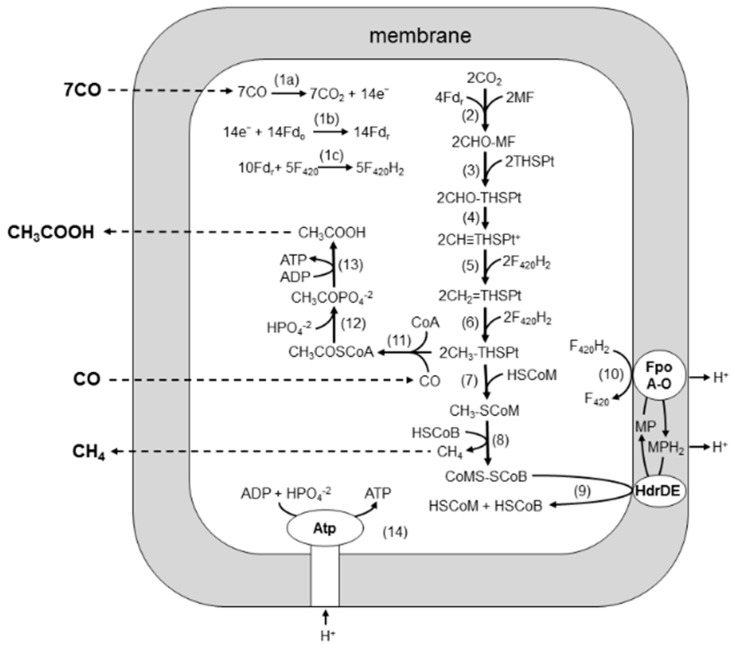
Pathway for conversion of CO to acetate and CH_4_ by *M. acetivorans*. See text. Fd_o_, oxidized ferredoxin; Fd_r_, reduced ferredoxin; F_420_, coenzyme F_420_; MF, methanofuran; THSPt, tetrahydrosarcinapterin; HSCoM, coenzyme M; HSCoB, coenzyme B; Fpo, F_420_H_2_ dehydrogenase complex; MP, methanophenazine; Hdr, heterodisulfide reductase. Reproduced from [16] with permission. Copyright (2006) National Academy of Sciences, USA.

Biochemical and quantitative proteomic analyses of *M. acetivorans* revealed a pathway for CO-dependent growth of *M. acetivorans* in which both acetate and CH_4_ are products [16,17] (Figure 3). In the pathway, electrons derived from the oxidation of CO (Reaction 1a–c) are used to reduce CO_2_ to a methyl group attached to THSPt (Reactions 2–6), similar to the pathway of obligate H_2_-utilizing CO_2_-reducing methanogens, which are the subject of several reviews recommended to the reader [18,19,20,21]. The CO dehydrogenase/acetyl-CoA synthetase CODH/ACS is proposed to oxidize CO-reducing ferredoxin, which donates electrons in the first reductive step (Reaction 2). It is possible that ferredoxin also donates electrons to FpoF, catalyzing the reduction of coenzyme F_420_ to F_420_H_2_ (Reaction 1c) that donates electrons to complete the reduction of CO_2_ to the methyl level (Reactions 5 and 6). Indeed, it was recently shown that the FpoF of *Methanosarcina mazei* catalyzes reaction 1c [22] and a homolog in *M. acetivorans* (encoded by MA3732) is upregulated 11-fold in CO- *vs.* acetate-grown cells [16]. The methyl group of methyl-THSPt is reduced to CH_4_ by reactions common to all methanogenic pathways (Reactions 7–9), albeit with two exceptions. First, a membrane-bound complex (FpoA-O) oxidizes F_420_H_2_ and transfers electrons to HdrDE mediated by MP, which generates a proton gradient (Reactions 9 and 10) driving ATP synthesis (Reaction 14). Second, transfer of the methyl group of methyl-THSPt to HS-CoM (Reaction 7) is thought to be catalyzed by two enzymes, the membrane-bound Mtr complex coupled to Na^+^ translocation, common to all methanogenic pathways; and a soluble enzyme (CmtA) unique to the CO-dependent CO_2_ reduction pathway [23]. Acetate is a prominent product in addition to CH_4_. Levels of CODH/ACS, phosphotransacetylase, and acetate kinase are prominent in CO-grown cells [16], supporting a route to acetate wherein CODH/ACS synthesizes acetyl-CoA with the methyl group of methyl-THSPt, CO, and CoA-SH (Reaction 11), which are further converted to acetate by phosphotransacetylase and acetate kinase (Reactions 12 and 13) with the production of ATP. Thus, ATP is synthesized via both substrate level and chemiosmotic mechanisms.

*A. fulgidus* uses essentially the same pathway during CO-dependent growth, employing similar enzymes and electron carriers [24,25,26,27,28], except for conversion of acetyl-CoA to acetate, which is catalyzed by either an AMP- or ADP-forming acetyl-CoA synthetase [29,30,31]. *A. fulgidus* does not produce methane, and acetate is the sole product in the absence of sulfate.

## 3. Acetate Utilization

### 3.1. Acetotrophic Energy-Converting Pathways Producing Methane

**Figure 4:**
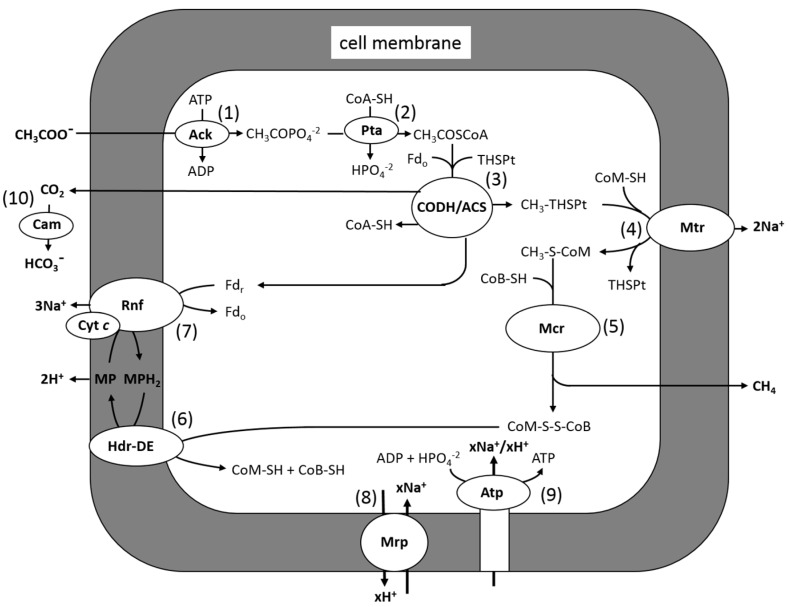
Pathway of aceticlastic methanogenesis in *M. acetivorans*. Ack, acetate kinase; Pta, phosphotransacetylase; CoA-SH, coenzyme A; THSPt, tetrahydrosarcinapterin; Fdr, reduced ferredoxin; Fdo, oxidized ferredoxin; Cdh, CO dehydrogenase/acetyl-CoA synthase; CoM-SH, coenzyme M; Mtr, methyl-THSPt:CoM-SH methyltransferase; CoB-SH, coenzyme B; MP, methanophenazine; Hdr-DE, heterodisulfide reductase; Rnf, Rnf complex; Mrp, Mrp complex; Atp, ATP synthase. Modified from [39].

Methane-producing species from the genera *Methanosarcina* and *Methanosaeta* are the only known acetoclastic genera in the domain *Archaea*. The majority of studies on the acetoclastic pathway have involved *Methanosarcina* species, for which *M. acetivorans* is a model (Figure 4). Carbon transfer reactions in the pathway can be divided into two parts: (1) Reactions 1–3: activation to acetyl-CoA and cleaving the C-C bond of the acetyl group, yielding CH_3_-THSPt and CO_2_, which are unique to the pathway; and (2) Reactions 4–6: reducing the methyl group of CH_3_-THSPt to CH_4_, which is common to all methanogenic pathways. Methanogenesis, and by inference reactions common to all methanogenic pathways, is thought to have evolved soon after the origin of life, approximately 3.75 billion years ago [32,33]. However, evolution of the acetoclastic pathway is proposed to have evolved approximately 250–300 million years ago during the end-Permian carbon cycle that contributed to the mass extinction of that period [34]. Reactions 2 and 3, catalyzed by acetate kinase and CO dehydrogenase/acetyl-CoA synthase CODH/ACS, are thought to be ancient enzymes evolving soon after the origin of life [35,36]. Homologs of acetate kinase and phosphotransacetylase are key enzymes in energy-yielding pathways of fermentative and acetogenic species from the domain *Bacteria*, converting acetyl-CoA to ATP and acetate. These observations are consistent with the evolution of the aceticlastic pathway by horizontal gene transfer of enzymes catalyzing Reactions 1–3 from the domain *Bacteria* and grafting on to Reactions 4–6, common to more ancient methanogenic pathways. Reactions 4 and 5, and the enzymes catalyzing them, are the subject of recent reviews that are recommended to the reader [37,38].

The first crystal structure for any acetate kinase was from the acetoclastic methanogen *Methanosarcina thermophila*, which revealed properties suggesting that the enzyme is the founding member of the Acetate and Sugar Kinase/Hsc70/Actin (ASKHA) superfamily of phosphotransferases [35]. Structural and biochemical investigations established a direct in-line mechanism in which the carboxylate anion attacks the γ-phosphate of ATP enabling transfer of the phosphate group [40,41,42,43,44,45,46,47,48]. The first crystal structure of any phosphotransacetylase was also from *M. thermophila*, the structural and biochemical analyses of which indicate a mechanism that proceeds through base-catalyzed abstraction of the HS-CoA thiol proton and subsequent nucleophilic attack of ^−^S-CoA on the carbonyl carbon of acetyl phosphate [49].

The CODH/ACS complex [50,51,52,53,54,55,56,57,58,59] cleaves the C-C and C-S bonds of acetyl-CoA, transferring the methyl group to THSPt and oxidizing the carbonyl group to CO_2_ with transfer of electrons to ferredoxin [60,61,62] (Reaction 3). The same enzyme complex functions in reverse to synthesize acetyl-CoA for cell carbon in CO_2_-reducing methanogens and oxidizes exogenous CO in the pathway of CO conversion to CH_4_, CO_2_, and acetate in *M. acetivorans*. Of ancient origin, primitive ancestors of CODH/ACS likely played a central role in the early evolution of life [36,63,64]. Although a two-subunit enzyme has been purified and characterized from an acetate-utilizing species of the genus *Methanosaeta* [54,57,58,59], the majority of mechanistic studies have been with the five-subunit (αβγδε) complexes from the acetate-utilizing species *M. thermophila* and *Methanosarcina barkeri*. The complexes are resolvable into three components containing the αε, γδ, or β subunit(s) [65]. The αε component catalyzes the oxidation of CO and reduction of ferredoxin [60,66]. The crystal structure from *M. barkeri* shows a α2ε2 arrangement with the α subunit containing four 4Fe-4S clusters and a NiFe3S4 cluster bridged to an exogenous iron atom called the “C” cluster [67]. Two of the 4Fe-4S clusters are postulated to function in electron transport from the active site “C” cluster to ferredoxin. The structure suggests coupling between CO bound to the nickel and H_2_O/OH− bound to the exogenous iron in the C=O bond-forming step leading to the oxidized product CO_2_. The structure also shows a gas channel extending from the “C” cluster to the protein surface with the potential to interface with the β component containing the “A” cluster, thus catalyzing acetyl-CoA cleavage and carbonyl group conversion to CO [68,69,70,71]. Although the structure is unknown, spectroscopic investigations indicate that the “A” cluster is comprised of a 4Fe-4S center bridged to a binuclear Ni-Ni site [70,72], similar to the homolog from an acetate-producing species from the domain *Bacteria* that synthesizes acetyl-CoA [73]. A mechanism is proposed in which transfer of an electron from “C” to “A” maintains the reduced catalytically active Ni(I) redox state of “A” [74]. Both kinetic and EPR approaches support the fact that alterations in the Ni coordination environment of the “A” cluster promote C−C bond cleavage, dependent on changes in the protein conformation from the open to closed state [75,76]. Moreover, CO is proposed to be an inhibitor of C-C bond cleavage; thus, control over C−C bond cleavage in concert with containment of CO in the gas channel explains the requirement for tight coupling of the decarbonylation reaction for efficient transfer of CO to “C” for oxidation [75]. The γδ component transfers the methyl group of acetyl-CoA to THSPt, involving a corrinoid cofactor and an iron-sulfur cluster [55,77,78,79], although it has yet to be determined which of the two subunits interact with THSPt. Spectroscopic EPR analyses indicate that the corrinoid cofactor is maintained in the base-off state with a $E_{0}^{'}$ of −486 mV for the Co^2+/1+^ couple that facilitates reduction of Co^2+^ to Co^1+^ required for methylation of the corrinoid [77]. The analysis also identified a 4Fe-4S cluster with a midpoint potential close to the Co^2+/1+^ couple, suggesting that the cluster is involved in reducing Co^2+^. 

The conversion of acetate to CH_4_ and CO_2_ provides only a marginal amount of energy available for growth (ΔG^0^*'* = −36 kJ/CH_4_) that is spent on the ATP consumed in the activation to acetyl-CoA (Figure 4), which illustrates the importance of cells maximizing thermodynamic efficiency. A theoretical analysis of acetate-grown *M. barkeri* indicates that transfer of CH_4_ and CO_2_ into the gaseous phase contributes to the driving force of growth [80]. Thus it has been proposed that a carbonic anhydrase (Cam) from *M. thermophila* is located outside the cell membrane, where it converts CO_2_ to membrane-impermeable HCO_3_^−^ (Reaction 10, Figure 4), thereby facilitating removal of CO_2_ from the cytoplasm [81]. Cam from *M. thermophila* is the archetype of an independently evolved class (γ class) of carbonic anhydrases that contains Fe^2+^ in the active site, contrary to all prokaryotic carbonic anhydrases, which contain zinc [82,83]. Structural and biochemical analyses [84] support a two-step ping pong mechanism, shown in Equations (6) and (7), where E represents enzyme residues, M is metal, and B is the buffer.

(6a) E-Fe^2+^-OH^−^ + CO_2_ = E-Fe^2+^-HCO_3_^−^

(6b) E-Fe^2+^-HCO_3_^−^ + H_2_O = E-Fe^2+^-H_2_O + HCO_3_^−^

(7a) E-Fe^2+^-H_2_O = H^+^-E-Fe^2+^-OH^−^

(7b) H^+^-E-Fe^2+^-OH^−^ + B = E-Fe^2+^-OH^−^ + BH^+^

In Step 1 a lone pair of electrons on the metal-bound hydroxide attacks CO_2_-producing metal-bound bicarbonate (Equation (6a)), which is subsequently displaced by water (Equation (6b)). In Step 2 a proton is extracted from the metal-bound water (Equation (7a)), and then transferred to the buffer (Equation (7b)).

ATP is synthesized by a chemiosmotic mechanism. Ferredoxin accepts electrons derived from the oxidation of the carbonyl group of acetyl-CoA by CODH/ACS in both H_2_-dependent and H_2_-independent acetotrophic methanogens. Both types also obtain energy for growth by coupling electron transfer from ferredoxin to the heterodisulfide CoM-S-S-CoB, with translocation of ions generating a gradient that drives ATP synthesis catalyzed by an A_1_A_0_-type ATP synthase [85,86,87,88]. The reduced ferredoxin of H_2_-dependent species donates electrons to a membrane-bound hydrogenase (Ech), evolving H_2_ and translocating protons [89,90,91]. It is proposed that a hydrogenase (Vho) reoxidizes H_2_ and donates electrons to a quinone-like electron carrier called methanophenazine (MP) [92]. The MP donates electrons to the heterodisulfide reductase HdrDE concomitant with translocation of two protons contributing to the gradient. An additional two protons are translocated by the Vho. However, most acetotrophic methanogens are H_2_ independent [93,94,95,96,97,98,99,100,101,102,103,104] and likely evolved a different strategy for oxidizing ferredoxin and reducing CoM-S-S-CoB typified by *M. acetivorans* [105,106] (Figure 4). The genome does not encode a functional Ech; instead, acetate-grown cells preferentially synthesize a Rnf complex [87] similar to the six-subunit Rnf complexes in microbes from the domain *Bacteria* [107,108,109,110,111,112,113]. A Δ*rnf* strain is unable to grow with acetate, confirming that the complex is essential [114]. Unlike all other characterized Rnf complexes, the contiguous genes encoding the six-subunit core complex from *M. acetivorans* are co-transcribed with a gene encoding a multiheme cytochrome *c* abundant in membranes of acetate-grown cells [87]. A topology model [39] predicts roles for each of the six core subunits and cytochrome *c* wherein MP mediates electron transfer between cytochrome *c* and HdrDE, translocating a pair of protons [39]. It has been shown that the Rnf complex translocates Na^+^ (Reaction 7), joining the Na^+^ translocating methyl transfer (Reaction 4) [114]. Thus, both Na^+^ and H^+^ gradients are generated during growth with acetate. Notably, the A_1_A_0_-type ATP synthase of *M. acetivorans* is dependent on both Na^+^ and H^+^ gradients [115]. A multisubunit Na^+^/H^+^ antiporter (Mrp) is proposed to adjust the Na^+^/H^+^ ratio (Reaction 8) optimal for ATP synthesis by the A_1_A_0_-type ATP synthase (Reaction 9) [116].

A genome-wide analysis of *Methanosaeta thermophila* has revealed genes encoding enzymes catalyzing reactions in the pathway of acetate to CH_4_ identical to *Methanosarcina* species except for the activation of acetate to acetyl-CoA, which is catalyzed by an AMP- and PP_i_-forming acetyl-CoA synthetase [117,118,119,120]. The synthetase has a several-fold lower *K*_m_ for acetate (0.4 mM) than acetate kinase from *M. thermophila* (22 mM) [118,121], a result consistent with *Methanosaeta* species dominating over *Methanosarcina* species in environments where acetate is in low concentrations. Genes encoding Ech and Rnf are absent in the genome of *Methanosaeta*, suggesting an unknown alternative electron transport pathway and mechanism for energy conservation [117].

### 3.2. Acetotrophic Energy-Converting Pathways Reducing Exogenous Electron Acceptors

Acetate-utilizing prokaryotes from the domain *Archaea* also obtain energy by anaerobic respiration. *Ferroglobus placidus* and *Geoglobus ahangari* are hyperthermophiles growing at 85 °C by oxidizing acetate to CO_2_, only with Fe(III) serving as the electron acceptor [122]. *Geoglobus acetivorans* is another hyperthermophile growing optimally at 81 °C and utilizing acetate in addition to formate, pyruvate, fumarate, malate, propionate, butyrate, succinate, glycerol, stearate, palmitate, peptone, and yeast extract as electron donors for Fe(III) reduction. The organism is also able to grow with H_2_ as the electron donor and Fe(III) as an electron acceptor without the need for organic substances [123]. Hyperthermophilic *Thermococcus* species have also been implicated in oxidizing acetate and reducing Fe(III) [124].

## 4. Concluding Remarks

Although heterotrophic acetate-producing hyperthermophiles are abundantly documented, no hyperthermophilic acetoclastic methanogen has been described that presents a disconnect in the ecology of these two metabolic groups for which cognate mesophiles function syntrophically in anaerobic microbial food chains, converting complex biomass to CH_4_. One possibility is that hyperthermophilic temperatures are a thermodynamic barrier to the conversion of acetate to CH_4_. Additionally, although it appears that heterotrophic organisms from the domain *Archaea* (Thaumarchaea) proliferate in mesothermal anaerobic environments [125], isolates and details of their metabolism are largely unknown. Thus, the finding that anaerobic respiratory acetate-oxidizing species are found in hyperthermophilic environments suggests the possibility that acetate-producing heterotrophs like *P. furiosus* supply acetate in a two-component microbial food chain converting complex organics to CO_2_.
